# Is there a directional relationship between windswept hips and scoliosis in children with cerebral palsy?

**DOI:** 10.1186/s12891-025-09355-8

**Published:** 2025-11-28

**Authors:** Elisabet Rodby-Bousquet, Hans Tropp, Atli Agustsson

**Affiliations:** 1 Department of Clinical Sciences Lund, Orthopaedics, Lund University, Lund, Sweden; 2 Centre for Clinical Research, Region Västmanland, Västerås, Sweden; 3 Department of Women’s and Children’s Health, Karolinska Institutet, Neuropaediatrics, Stockholm, Sweden; 4 Department of Orthopedic Surgery, Department of Biomedical and Clinical Sciences, Linköping University, Linköping, Sweden; 5 Centre for Medical Image Science and Visualization (CMIV), Linköping University, Linköping, Sweden; 6 Research Centre of Rehabilitation and Movement Science, Department of Physiotherapy, University of Iceland, Reykjavik, Iceland

**Keywords:** Cerebral palsy, Children, Contracture, Hip joint, Scoliosis, Windswept hips

## Abstract

**Background:**

The purpose of this study was to analyse if there is a relationship between the directions of windswept hip deformity and scoliosis for children with cerebral palsy (CP) and to explore potential associations with spasticity, contractures and hip displacement.

**Methods:**

A cross-sectional registry-based study was conducted using data from the combined Swedish CP Follow-up Program and National Registry, including children aged 0–18 years across all levels of the Gross Motor Function Classification System. Prevalence and direction of windswept hip deformity was assessed based on calculations of passive range of motion in abduction, internal and external rotation of the hips. Scoliosis was defined as either a moderate or severe curve at clinical examination or a radiographic Cobb-angel of ≥ 20°. The direction of scoliosis was determined by the convexity of the spinal curve.

**Results:**

A total of 4,453 children (mean age 10.2 years [SD 4.8]) were included. In total 774 (17.4%) had scoliosis (*n* = 503) or windswept hips (*n* = 413). Among these141 had both scoliosis and windswept hips allowing for analyses of directional relationships. The scoliosis and windswept hip deformity were going in opposite directions in 75 of the 141 children, and in the same direction in 66 children. More children with scoliosis and windswept hips to the same side had a contralateral hip displacement (*p* < 0.001). Whereas children with deformities in opposite directions more frequently had a hip displacement contralateral to the windswept hip deformity. The direction of the scoliosis was associated with a contralateral hip displacement (*p =* 0.020). The direction of the windswept hip deformity was associated with a contralateral hip displacement (*p* < 0.001), hip flexion contracture (*p* = 0.002) and spasticity in the hip adductors on the opposite side (*p* < 0.001).

**Conclusions:**

There is no clear directional relationship between windswept hips and scoliosis in children with CP. However, the direction seems to be highly associated with the location of hip displacement, hip flexion contracture and spasticity of the hip adductors. These findings suggest that preventing and treating contractures in the lower extremities, along with active hip surveillance, may help reduce the development of both scoliosis and windswept hip deformity.

## Introduction

Children with cerebral palsy (CP) are typically born without musculoskeletal problems, but contractures and deformities of the spine and hips may develop as they age [1]. Alterations in muscle volume and growth can occur as early as infancy [2]. The muscles in these children contain fewer satellite cells and energy-generating organelles which may affect muscle growth and contribute to contracture development [3, 4]. Contractures most commonly develop first in the knee [5], followed by contractures of the hip [6] and may progress to the development of a neuromuscular scoliosis [7].

The prevalence of scoliosis in children with CP ranges from 11 to 41% [8, 9] depending on factor such as age, cohort characteristics and severity of the spinal curve. Several risk factors have been identified for the development and progression of neuromuscular scoliosis, including severe motor impairment with trunk instability, knee contracture, epilepsy, increasing age and female sex [7, 10]. The incidence of scoliosis increases with age and GMFCS level, with a significantly higher risk for individuals at GMFCS IV and V [7, 9, 11] who typically spend more time sitting in wheelchairs.

Windswept hip deformity develops in approximately 16% of the children with CP [1]. This deformity is characterised by abduction and external rotation of one hip, accompanied by adduction and internal rotation of the other hip. Some researchers attribute this adaptation to asymmetrical muscle tone and severe spasticity [12, 13], while others propose that it results from altered hip joint morphology and reduced stability [14]. There are indications suggesting that early treatment of contractures in the lower extremities, may reduce the incidence of windswept deformity, supporting the latter hypothesis [15]. It is likely that both mechanisms play a role, as the muscle imbalance around the morphological altered hip joint can contribute to hip pathologies [16, 17].

Knee contracture has been identified as a risk factor for the development of both windswept hips and scoliosis [7, 18]. Although windswept hips typically develop before the onset of scoliosis in most children with CP [1], the directional relationship between these deformities remains unclear [19, 20]. Porter et al. [19] found that windswept hips were usually directed in the opposite direction to the convexity of the scoliosis curve. In contrast, both Lonstein et al. [8] and Young [9] reported no correlation between the direction of the scoliosis curve and the direction of the windswept hips.

Most CP patients with scoliosis present a long and regular thoracolumbar curve down to the pelvis [21]. Pelvic obliquity may result from asymmetric muscles connecting trunk and pelvis but can also be related to asymmetry of the pelvic and hip muscles [21]. The purpose of this study was to analyse the relationship between the direction of the windswept hip deformity and the direction of the scoliosis in children with CP and explore potential associations with spasticity, contractures and hip displacement.

## Methods

A cross-sectional registry study was performed using data from the most recent examination of all children aged 0–18 years, registered in the combined Swedish Cerebral Palsy Follow-up Program and National Registry (CPUP) from 2018 to 2021. This registry holds standardised data collected at annual examinations by physiotherapists and occupational therapists working with these children, and includes over >95% of all children with CP in Sweden [22]. The study was approved by the Swedish Ethical Review Authority (2023-01723-01). Caregivers of all the children have given informed consent to research based on the data held in the registry.

### Classifications and measurements

The CP diagnosis was based on the inclusion and exclusion criteria by the Surveillance of Cerebral Palsy in Europe [23]. Neurological subtype was classified into either ataxic, dyskinetic, spastic CP or mixed type of CP. Gross motor function was classified using the expanded and revised version of the Gross Motor Function Classification System (GMFCS) [24]. Children were included in the study if data were available for both outcome variables of scoliosis and windswept hip deformities.

Windswept hip deformity and the direction of the hips were calculated based on passive range of motion (ROM) of abduction, internal and external rotation of the hips with a difference of at least 50% between the left and right sides, following Persson-Bunke’s method [25]. ROM of the hip joints was measured using standardised goniometric procedures, with the child in a supine position for hip abduction measurements and in a prone position for internal and external hip rotation. Hip and knee extension were measured in supine position and a contracture was defined as a flexion contracture of at least 10°. Hip displacement with a migration percentage (Reimers’ index) of at least 40%, was calculated from standardized anteroposterior pelvic radiographs.

Clinical spinal examination was performed with the child seated upright on a plinth using the Adam´s forward bend test. The severity of scoliosis was graded as moderate (obvious curve present in both upright and forward bending) or severe (pronounced curve that prevents upright position without external support). This method has been reported to have high interrater reliability, as well as high sensitivity and specificity when compared to radiographic Cobb angles of ≥ 20° [26]. Cobb angles were categorised as ‘moderate’ (20–39°), or ‘severe’ scoliosis (*≥* 40°). Additionally, a history of previous spinal surgery was categorized as equivalent to severe scoliosis. The direction of the curve was noted as convex right and/or left depending on the shape of the curve.

The Modified Ashworth scale (MAS) [27] was used to evaluate increased muscle tone/spasticity of the hip adductors and hip flexors. MAS 2, 3, and 4 were grouped together and considered as increased tone/spasticity. Spasticity (MAS 2 to 4), on both sides was treated as bilateral spasticity. Conversely, MAS rated as 2–4 on one side only, was treated as unilateral spasticity, and the affected side was documented.

### Statistical analyses

Categorical data was described using frequencies and percentages, *n* (%), while continuous data were reported as means with accompanying standard deviations (SDs). Age was grouped into six categories: 0–3, 4–6, 7–9, 10–12, 13–15 and 16–18 years old. Pearson’s χ^2^-test and the χ^2^-test for trend were used for tests of differences between categorical variables. Fisher’s exact test was used for small groups. IBM SPSS Statistics 28 was used for statistical analyses, with p-values < 0.05 considered statistically significant.

## Results

There were 4,453 children with CP (0–18 years, mean age 10.2 [SD 4.8]) across all GMFCS levels reported into the registry (Table 1). Among these, 774 (17.4%) had scoliosis (*n* = 503) or windswept hips (*n* = 413). The prevalence of scoliosis and windswept hips increased with higher GMFCS levels and varied significantly between CP subtypes, with the highest proportions observed in children with dyskinetic CP. Additionally there was also a slightly higher proportion of girls with scoliosis (12.4%) compared with boys (10.5%) (Table 1). In total 141 (3.1%) had both scoliosis and windswept hips, allowing us to analyse the directional relationships between the two deformities.

**Table 1:** Number and percentages of all children with scoliosis and windswept hips across sex, gross motor function classification system (GMFCS) levels I to V and CP subtype

		All children	All with Scoliosis		All with Windswept		Scoliosis & Windswept	
		*n (%)*	*n (%)*	*P*	*n (%)*	*P*	*n (%)*	*P*
Sex	Boys	2602 (58.4)	273 (10.5)	0.045	238 (9.1)	0.727	79 (3.0)	0.492
	Girls	1851 (41.6)	230 (12.4)		175 (9.5)		62 (3.3)	
GMFCS	I	2002 (45.0)	26 (1.3)	< 0.001	76 (3.8)	< 0.001	1 (0.05)	< 0.001
	II	710 (15.9)	34 (4.8)		55 (7.8)		5 (0.7)	
	III	370 (8.3)	28 (7.6)		26 (7.0)		6 (1.6)	
	IV	634 (14.2)	117 (18.5)		87 (13.7)		30 (4.7)	
	V	737 (16.6)	298 (40.4)		169 (22.9)		99 (13.4)	
Subtype	Ataxic	172 (3.9)	9 (5.2)	< 0.001	3 (1.7)	< 0.001	1 (0.6)	0.003
	Dyskinetic	507 (11.5)	92 (18.1)		68 (13.4)		25 (4.9)	
	Spastic	3487 (79.4)	367 (10.5)		324 (9.3)		113 (3.2)	
	Mixed	228 (5.2)	35 (15.4)		18 (7.9)		2 (8.8)	
	Missing	59						
Total		4453 (100)	503 (11.3)		413 (9.3)		141 (3.1)	

### Direction of scoliosis and windswept hips

Direction of the scoliosis was reported for 405 of the 503 children. Among them, 186 children (46%) had a curve convex to the right, and 219 children (54%) to the left. In contrast, the direction of windswept hips showed the opposite pattern, with 218 children (53%) having windswept to the right and 195 children (47%) to the left.

### Associations between scoliosis, spasticity, contractures and hip displacement

Of the 405 children with reported scoliosis direction, 76 children (19%) had spasticity (MAS 2–4) in the hip adductors (56 bilateral, 20 unilateral) and 38 (9.4%) had spasticity in the hip flexors (24 bilateral, 14 unilateral). Additionally, 88 children (22%) had a hip flexion contracture of at least 10° (52 bilateral, 36 unilateral), and 252 children (62%) had a knee flexion contracture of at least 10° (185 bilateral, 67 unilateral). Moreover, 81 children (20%) had a hip displacement with a migration percentage (Reimers index) of at least 40% (14 bilateral, 67 unilateral). Neither the side of spasticity nor the side of the contracture seemed to be associated with the direction of scoliosis. However, the direction of the scoliosis was more frequently associated with a contralateral hip displacement (45 vs. 22) (*p* < 0.001) (Table 2).

**Table 2:** Direction of scoliosis and windswept hips respectively, in relation to sides of spasticity (hip adductors and flexors), contractures (hip and knee) and hip displacement (Reimers Index, RI)

		Direction	Scoliosis		Windswept	
			*n*	*p*	*n*	*p*
Spasticity MAS 2–4	Hip adductors	Same side	13	0.609	9	0.002
		Opposite side	7		27	
		Bilateral	56		53	
	Hip flexors	Same side	5	0.773	8	0.780
		Opposite side	9		11	
		Bilateral	24		20	
					39	
Contracture *≥* 10º	Hip extension	Same side	19	0.789	13	0.018
		Opposite side	17		28	
		Bilateral	52		68	
	Knee extension	Same side	27	0.147	31	0.691
		Opposite side	40		30	
		Bilateral	185		188	
Hip displacement	RI *≥* 40%	Same side	22	0.020	11	< 0.001
		Opposite side	45		43	
		Bilateral	14		8	
Total			405		413	

* Fishers’s exact test

### Associations between windswept, spasticity, contractures and hip displacement

Among the 413 children with windswept hip deformity, 89 children (22%) had spasticity in the hip adductors (MAS 2–4) (53 bilateral, 36 unilateral), and 39 children (9.4%) had spasticity in the hip flexors (20 bilateral, 19 unilateral). In addition, 109 children (26%) had a hip flexion contracture of at least 10° (68 bilateral, 41 unilateral) and 249 children (60%) had a knee flexion contracture (188 bilateral, 61 unilateral). Additionally, 62 children (15%) had a hip displacement with a Reimers index of at least 40% (8 bilateral, 54 unilateral). No directional differences were observed between the windswept hip deformity and spasticity of the hip flexors or knee contracture. However, the direction of the windswept hip deformity was more frequently associated with a contralateral hip flexion contracture (28 vs. 13) (*p* = 0.018), spasticity in the hip adductors on the opposite side (27 vs. 9) (*p* = 0.002) and a contralateral hip displacement (43 vs. 11) (*p* < 0.001).

### Directional relationship between scoliosis and windswept hip deformity

The scoliosis and windswept hip deformity were going in opposite directions in 75 of the 141 children (53%), and in the same direction in 66 children (47%). Slightly more children had scoliosis to the left and windswept hips to the right. The directional relationship between scoliosis and windswept hips was similar across sexes, ages, GMFCS levels and CP subtypes (Table 3). However, when scoliosis and windswept hip deformity were in the same direction, they were associated with a contralateral hip displacement (18 vs. 0) (*p* < 0.001). Also, when the two deformities were in opposite directions, the direction of the windswept was more frequently associated with a hip displacement on the contralateral side (10 vs. 6) (Table 3).

**Table 3:** Direction of scoliosis and windswept hips across sex, gross motor function classification system (GMFCS) levels I to V, CP subtype and hip displacement > 40%

		Same direction		Opposite directions		
		Both Left	Both Right	Scoliosis Right	Scoliosis Left	
				**Windswept Left**	**Windswept Right**	
		***n*** ** (%)**	***n*** ** (%)**	***n (%)***	***n (%)***	P*
Sex	Boys	17 (21.5)	17 (21.5)	21 (26.6)	24 (30.4)	0.621
	Girls	16 (25.8)	16 (25.8)	11 (17.7)	19 (30.6)	
GMFCS	I	0 (0)	1 (100)	0 (0)	0 (0)	0.431
	II	0 (0)	2 (40.0)	2 (40.0)	1 (20.0)	
	III	3 (50.0)	0 (0)	0 (0)	3 (50.0)	
	IV	7 (23.3)	7 (23.3)	5 (16.7)	11 (36.7)	
	V	23 (23.2)	23 (23.2)	25 (25.3)	28 (28.3)	
Subtype	Ataxic	0 (0)	0 (0)	0 (0)	1 (100)	0.259
	Dyskinetic	2 (8.0)	7 (28.0)	6 (24.0)	10 (40.0)	
	Spastic	30 (26.5)	26 (23.0)	25 (22.1)	32 (28.3)	
	Mixed	1 (50.0)	0 (0)	1 (50.0)	0 (0)	
Hip displacement	RI > 40% Left	0 (0)	9 (52.9)	3 (17.6)	5 (29.4)	< 0.001
	RI > 40% Right	9 (52.9)	0 (0)	5 (29.4)	3 (17.6)	
Total		33 (23.4)	33 (23.4)	32 (22.7)	43 (30.5)	

* Fishers’s exact test

## Discussion

We were unable to establish a definitive relationship between the direction of windswept hip deformity and the direction of scoliosis in children with CP. In our study, the scoliosis and windswept hip deformity were in opposite directions in 53% of the children and in the same direction in 47%. However, we identified some directional differences related to spasticity of the hip adductors, contractures around the hip joint and hip displacement.

The prevalence of scoliosis was 11%, which is in line with previous research including moderate and severe curves identified through clinical examination or Cobb angles *≥* 20° at radiographic examination [9]. However, this prevalence was significantly lower than studies including Cobb angles of 10° [8]. Moderate and severe curves identified at clinical examination have demonstrated high sensitivity, specificity, positive and negative likelihood ratio when compared with radiographic Cobb angles of 20° [26]. The prevalence of windswept was 9%, when calculated as a 50% difference between the right and left side. This is slightly lower than the incidence reported in longitudinal studies [1] but consistent with prevalence reported for children with CP in cross-sectional studies [15]. The method used for these calculation has previously been described by Persson-Bunke [28] and reported in several studies [1, 29].

Our findings indicate that in slightly more than half of the children (53%) the scoliosis and windswept hip deformity were in opposite directions. This differs slightly from the study by Porter et al. [19], which reported a higher proportion (62%) of windswept hips in the opposite direction to the convexity of the scoliosis curve, based on a sample of children and adults 6 to 80 years at GMFCS level V. Young et al. [9] identified 15 children with scoliosis and windswept hips in opposite directions, compared to 11 children with the deformities in the same direction, though the difference was not statistically significant. Porter also noted a slightly higher proportion of scoliosis convex to the left (56%), which is consistent with the 54% observed in our study, and windswept hips to the right (57%) compared to the 53% we found.

Our findings indicate that spasticity of the hip adductors on one side are associated with a higher proportion of windswept hip deformities to the opposite side. This partially aligns with previous studies suggesting that windswept hip deformity can result from asymmetrical muscle tone and severe spasticity [12, 13]. Additionally, we found a higher proportion of windswept hip deformities to the opposite side of a hip flexion contracture or a hip displacement of at least 40% Also, the direction of the scoliosis was more frequently associated with a hip displacement on the opposite side. Flexion contractures of the lower limb have previously been identified as risk factors for both windswept hips and scoliosis [7, 18] although the directional relationship has not been evaluated. These findings suggest that the prevention and treatment of contractures in the lower extremities could potentially reduce the occurrence of both scoliosis and windswept hip deformity. Our findings highlight the importance of hip surveillance in addition to early prevention and management of spasticity and contractures in clinical practice, to reduce the risk of developing both scoliosis and windswept hip deformity in children with CP. This would be especially important from an early age and continuing into adulthood, as contractures and deformities tend to increase over time [1, 30].

### Limitations

This cross-sectional study can estimate differences and associations between variables at a single time point but not establish a causal relationship. Measurement errors are inherent in nearly all clinical assessments including goniometric measurement of the hip and knee joints. We selected a 10° cut-off for defining a flexion contracture of the hip or knee, as this threshold affects the biomechanical alignment and has been identified as a risk factor for windswept hips [31] and scoliosis in children with CP [7]. Spasticity of the hip adductors and hip flexors were rated according to the Modified Ashworth Scale (MAS). Although the MAS has moderate to high reported reliability for assessing spasticity in the lower extremities in children with CP [32], it remains the most widely used scale in clinical practice. To define spasticity, we only included MAS levels 2–4, indicating a more marked increase in muscle tone through most of the ROM. A limitation of our data was that scoliosis was classified only as moderate or severe, without distinguishing curve types (e.g., double thoracic–lumbar vs. long C-shaped curves). As these patterns may have different biomechanical implications, combining them could mask specific directional relationships. Another limitation is the absence of consistent clinical or radiographic measures of pelvic obliquity. As pelvic alignment is a critical biomechanical link between the spine and hips, it may act as a mediating or confounding factor in the relationship between scoliosis and windswept deformity. A notable strength of this study is that we included a total population of children with CP across all GMFCS levels.

## Conclusion

There was no clear directional relationship between scoliosis and windswept hip deformity in children with CP although they occurred in opposite directions in slightly more children. More children with scoliosis and windswept hips to the same side had a contralateral hip displacement. Whereas children with scoliosis and windswept hips in opposite directions more frequently had a hip displacement contralateral to the windswept hip deformity. Also, the direction of the scoliosis was more frequently associated with a contralateral hip displacement whereas the direction of the windswept hip deformity was associated both with a contralateral hip displacement, hip flexion contracture and spasticity in the hip adductors on the opposite side. These findings suggest that preventing and treating contractures in the lower extremities, along with active hip surveillance, could potentially reduce the development of both scoliosis and windswept hip deformity.

## Data Availability

The dataset analysed during the current study is part of the CPUP registry and is not publicly available due to ethical restrictions. However, anonymised data used in this study can be made available upon reasonable request from the corresponding author.

## Abbreviations

CP: Cerebral palsy
CPUP: Cerebral palsy follow up programme and registry
GMFCS: Gross motor function classification system
MAS: Modified ashworth scale
ROM: Range of motion
